# 4115 Fostering a learning environment to train and support versatile scientists who integrate science into real world operations of complex, dynamic health and public health systems

**DOI:** 10.1017/cts.2020.392

**Published:** 2020-07-29

**Authors:** Tony Kuo, Moira Inkelas, Onyebuchi A. Arah, Vladimir G. Manuel

**Affiliations:** 1 David Geffen School of Medicine at UCLA; 2 UCLA Fielding School of Public Health; 3 UCLA Health

## Abstract

OBJECTIVES/GOALS: The UCLA Clinical and Translational Science Institute’s Population Health Program is creating versatile scientists who can solve population health problems. This means building learning capability in health care and public health agencies, and fostering a cross-sector, outcomes-based, regional ecosystem for implementation and improvement. METHODS/STUDY POPULATION: A synthesis of achievements and lessons learned reveals the Program’s trajectory. It maps progress in science leading to sustainable interventions for target populations. PHP goals are predicated on networked team science, rather than disorganized assortment of individual studies and interventions, and emphasize design, modeling and iteration. Evolving metrics include network analysis to document collaborative impact; extent of integrating real-world application into systems science and learning system curriculum; legislative and institutional policies developed and adopted; evidence of system orientation, cross-sector focus, and implementation research in scientists’ portfolios; and demonstration of population health impact. Barriers offer the opportunity for iteration and improvement. RESULTS/ANTICIPATED RESULTS: The PHP has progressed in its envisioned shared university-public health stewardship of translation and transformation. Milestones included galvanizing activities such as annual regional dissemination, implementation, and improvement (DII) symposia and Public Health Science Summits; pre- and post-doctoral experiential learning of system science and learning system methods based in Los Angeles County Health Agency initiatives; development of a regional CTSA network for implementation science training; strengthened public health policy practice (e.g., establishing a new Office of Youth Diversion and Development); learning healthcare system capability; and prototypes of population learning systems focused on hypertension, food insecurity, tobacco/vaping, and complex care management. DISCUSSION/SIGNIFICANCE OF IMPACT: PHP is committed to advancing science for population health. Prototypes were an essential initial phase. New areas include use of methodological advances (e.g., artificial intelligence, rapid assessments) in health and public health systems; an academic home for full-time, population-focused clinicians; and social policy innovations.

